# Putative Mixotrophic Nitrifying-Denitrifying Gammaproteobacteria Implicated in Nitrogen Cycling Within the Ammonia/Oxygen Transition Zone of an Oil Sands Pit Lake

**DOI:** 10.3389/fmicb.2019.02435

**Published:** 2019-10-24

**Authors:** Jiro F. Mori, Lin-Xing Chen, Gerdhard L. Jessen, Sarah B. Rudderham, Joyce M. McBeth, Matthew B. J. Lindsay, Gregory F. Slater, Jillian F. Banfield, Lesley A. Warren

**Affiliations:** ^1^Department of Civil and Mineral Engineering, University of Toronto, Toronto, ON, Canada; ^2^Department of Earth and Planetary Sciences, University of California, Berkeley, Berkeley, CA, United States; ^3^Department of Geological Sciences, University of Saskatchewan, Saskatoon, SK, Canada; ^4^School of Geography and Earth Science, McMaster University, Hamilton, ON, Canada

**Keywords:** nitrifier denitrification, nitrogen cycle, oil sands, tailings deposits, shotgun metagenomics

## Abstract

Anthropogenically-impacted environments offer the opportunity to discover novel microbial species and metabolisms, which may be undetectable in natural systems. Here, a combined metagenomic and geochemical study in Base Mine Lake, Alberta, Canada, which is the only oil sands end pit lake to date, revealed that nitrification was performed by members from Nitrosomonadaceae, Chloroflexi and unclassified Gammaproteobacteria “MBAE14.” While Nitrosomonadaceae and Chloroflexi groups were relatively abundant in the upper oxygenated zones, MBAE14 dominated the hypoxic hypolimnetic zones (approximately 30% of total microbial communities); MBAE14 was not detected in the underlying anoxic tailings. Replication rate analyses indicate that MBAE14 grew in metalimnetic and hypolimnetic water cap regions, most actively at the metalimnetic, ammonia/oxygen transition zone consistent with it putatively conducting nitrification. Detailed genomic analyses of MBAE14 evidenced both ammonia oxidation and denitrification into dinitrogen capabilities. However, the absence of known CO_2_-fixation genes suggests a heterotrophic denitrifying metabolism. Functional marker genes of ammonia oxidation (*amo* and *hao*) in the MBAE14 genome are homologous with those conserved in autotrophic nitrifiers, but not with those of known heterotrophic nitrifiers. We propose that this novel MBAE14 inhabits the specific ammonia-rich, oxygen and labile organic matter-limited conditions occurring in Base Mine Lake which selectively favors mixotrophic coupled nitrifier denitrification metabolism. Our results highlight the opportunities to better constrain biogeochemical cycles from the application of metagenomics to engineered systems associated with extractive resource sectors.

## Introduction

The biogeochemical nitrogen cycle involves many microbially-catalyzed forward and reverse redox reactions that interconvert nitrate, nitrite, ammonia, nitric acid, nitrous oxide, and dinitrogen species. Over the last decade, culture-independent surveys for functional marker genes of ammonia oxidation such as ammonia monooxygenase (*amo*) have enabled identification of the potential for nitrification within microbial communities and in specific taxa previously unknown to nitrify. Further, metagenome-assembled genomes can reveal novel N metabolic pathways; for example, the complete ammonia oxidation (commamox) pathway able to oxidize ammonia to nitrate (Daims et al., 2015; van Kessel et al., 2015).

Microbial nitrification and denitrification have many contrastive aspects, i.e., nitrification is typically conducted by obligately aerobic chemolithoautotrophs, while denitrification is driven by anaerobic heterotrophs (Kuypers et al., 2018 and references therein). The distribution of these metabolic pathways may overlap spatially in environments where the oxic/anoxic boundary varies through time. Denitrification via autotrophic ammonia-oxidizing bacteria (AOB) (Poth and Focht, 1985; Shaw et al., 2006), as well as non-energy-generating ammonia oxidation coupled to denitrification via heterotrophs (heterotrophic AOB) (Kampschreur et al., 2009) can co-occur at low oxygen concentrations. An oxic/anoxic interface is thus an enticing environment to study the microbial ecology driving elusive N cycles (Paerl and Pinckney, 1996; Brune et al., 2000), where microorganisms may dynamically switch the direction of nitrogen compound redox transformations. Although previous culture-independent surveys indicate the ubiquitous existence of aerobic nitrifiers in natural environments, they rarely dominate communities, and often face competition for ammonia and oxygen with heterotrophs (Geets et al., 2006). However, they do succeed in ammonium-rich, aerated, and organic matter-limited conditions found in engineered systems such as wastewater treatment systems (Dionisi et al., 2002; Kuypers et al., 2018). Many recently discovered nitrifying bacteria have been found in anthropogenically-impacted environments. Anaerobic ammonia-oxidizing (anammox) bacteria were discovered in a wastewater treatment system (Jetten et al., 1998), the first isolated ammonia-oxidizing archaea (AOA) *Nitrosopumilus* spp. was isolated from a marine aquarium tank (Könneke et al., 2005) and the comammox *Nitrospira* spp. was isolated from a deep oil exploration well (Daims et al., 2015). Thus, the specific targeting of anthropogenic, ammonia-rich environments may reveal novel nitrifying players that may also be present in natural environments as members of the rare biosphere.

Unexpected microbial communities have been found in mining-impacted environments (Thavamani et al., 2017), where organisms may adapt complex life cycles, specialized nutrient uptake mechanisms and metabolic activities. Our research has focused on an engineered pit lake in the Athabasca oil sands region of north-eastern Alberta (Canada), where geochemical results evidence microbial nitrification occurring in the water cap of this aquatic mine reclamation landform (Risacher et al., 2018). Base Mine Lake (Syncrude Canada; Fort McMurray, AB, Canada) is the first full-scale demonstration of water capped tailings reclamation technology and was commissioned in 2012 as a demonstration of oil sands pit lakes (Dompierre et al., 2016). At commissioning, Base Mine Lake comprised ca. 45 m depth of fluid fine tailings (FFT) covered with an approximately 8 m freshwater and tailing porewater release cap. Previous findings showed a strong negative correlation between concentrations of water cap oxygen and concentrations of dissolved aqueous methane and ammonia, mobilized from the underlying FFT during summer stratification (Risacher et al., 2018). Thus, a trend of decreasing oxygen and increasing methane and ammonia concentrations with depth occurs within the water cap. However, methane was largely extinguished within the stratified lower hypolimnetic region of Base Mine Lake, while ammonia was detectable throughout the water cap, extending the possibility of nitrification higher up into the water cap region coincident with higher oxygen concentrations (Risacher et al., 2018). Further, the bulk organic carbon in this system is highly complex and less aerobically-biodegradable (Foght et al., 2017). Thus, despite relatively high DOC values (∼40 mg/L; Syncrude Canada Ltd., 2017), Base Mine Lake is a potential hotspot for microbial nitrification, as the constant supply of ammonia and limited labile organic matter may enable nitrifying microorganisms to outcompete copiotrophs.

We sampled Base Mine Lake microbial communities during mid-summer of 2015 and 2016 to identify the microorganisms involved in nitrogen cycle within this engineered system. 16S rRNA gene amplicon analysis was coupled with genome-resolved metagenomics to survey community compositions and to identify and characterize the nitrifiers present at both spatial and temporal scales. Determination of depth dependent water cap associated microbial community structure and function was complemented by geochemical characterization from the surface to the underlying FFT.

## Materials and Methods

### Site Description

**FIGURE 1:**
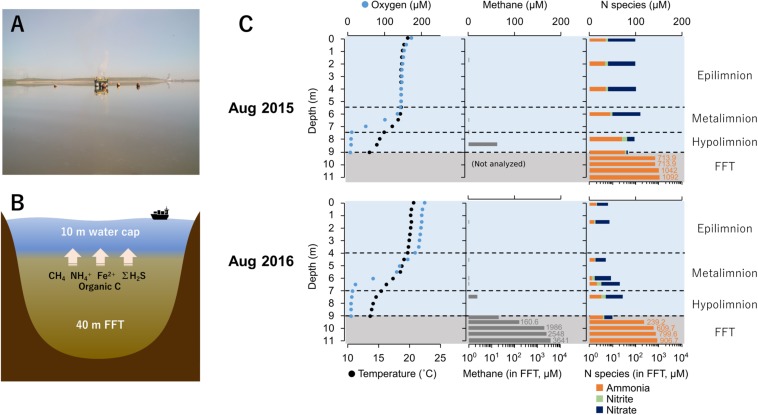
**(A)** Base mine lake identifying the sampling platform where samples were collected (August 2016). **(B)** Cross-section model of Base Mine Lake showing main reductants released from the ∼40 m layer of fluid fine tailings (FFT). **(C)** Base Mine Lake physicochemical and geochemical water cap and shallow (2 meter) of underlying FFT profiles for August 2015 and 2016. Dissolved oxygen concentration (μM) and temperature (°C) in the left column, dissolved aqueous methane concentration (μM) in the center column, and concentrations of three N redox species (ammonia, nitrite and nitrate; μM) in the right column. Nitrogen species concentrations for 2015 were obtained from Syncrude’s Base Mine Lake monitoring program (depths 0, 2, 4, 6, 8, 9 m) collected on August 10th, 2015. Dissolved aqueous methane and ammonia concentrations in the FFT were measured on August 5th 2015 and August 13th 2016.

Base Mine Lake (57° 0′ 38.88″N, 111° 37′ 22.44″W) is a 7.8 km^2^ engineered lake (Figure 1A) located 40 km north of Fort McMurray, Alberta, Canada. Tailings waste from extraction and processing of bitumen was pumped into a mined out pit from 1995 until 2012 (Dompierre et al., 2016; Foght et al., 2017), and the solids allowed to settle out, forming semi-consolidated FFT. FFT is composed of 50–60 wt% solids (clays, silts, and sands), containing ca. 1–5% residual bitumen (Chalaturnyk et al., 2002; Dompierre et al., 2016). In December 2012, FFT inputs ceased and Base Mine Lake was commissioned as the first water-capped tailings technology demonstration reclamation project. Typical oil sands tailings ponds are anoxic within the first meter of water depth, driven by oxidation of hydrogen sulfide (ΣH_2_S), methane (CH_4_), and ammonia (NH_4_^+^) generated by anaerobic microbial activity (Foght et al., 2017). Base Mine Lake has a deeper water cap (∼10 m) relative to the typical <5 m depth water cap in tailings ponds (Figure 1B), to increase the distance between FFT mobilized reductants such as methane, hydrogen sulfide and ammonia from the upper water cap. This depth separation increases the likelihood that a stable oxic zone will remain within the upper water column to sustain macrofauna; a criterion for success of this reclamation technology.

### Physicochemical Profiling, Sample Collection, and Chemical Analyses for Base Mine Lake

Water cap physico chemical profiling and sampling were conducted by boat during mid-summer stratification in 2015 and 2016 (August 18–20th and August 2–5th respectively), as a part of the larger study by Risacher et al. (2018; June–August 2015 and May–September 2016). Temperature, pH, dissolved oxygen, and conductivity measurements were conducted using a YSI Professional Plus 6-Series Probe (Xylem, Rye Brook, NY, United States) at 0.5–1.0 m intervals from surface to the FFT-water interface (approximately 9.0 m in 2015 and 9.3 m in 2016, following consolidation of the FFT). Water samples were collected using a 6.2 L Van Dorn bottle sampler (Waster Mark, Forestry Supplies, Jackson, MS, United States) from each thermally stratified region, i.e., epilimnion, metalimnion and hypolimnion, as identified by the physicochemical profile (4 depths in Aug 2015 and 7 depths in Aug 2016 respectively, Figure 1C). Samples for dissolved aqueous CH_4_, ΣH_2_S, Fe^2+^, and nitrogen redox species (NO_3_^–^, NO_2_^–^, and NH_4_^+^) as well as SO_4_^2–^, were analyzed using a HACH portable spectrophotometer (DR/2800, HACH, Loveland, CO, United States) as previously described by Risacher et al. (2018). At the same depths, ca. 1.5 L water was collected for microbial DNA extraction and filtered on board (see details below). Total- and dissolved organic carbon (TOC/DOC) samples were collected into the carbon-free glass bottles and stored at −20°C. Samples were analyzed using Total Organic Carbon Analyzer TOC-L_*CPH/CPN*_ (Shimadzu, Japan; DOC was determined after passing samples through 0.45 μm glass fiber filters).

Physicochemical profiling and sampling of the FFT were carried out on August 5th, 2015 and August 13th, 2016 at the location as the water cap sampling campaigns by Risacher et al. (2018). The FFT samples were collected into 250 mL high density polyethylene bottles using a custom-built fixed interval sampler for collecting FFT at 0.10 m vertical spacing over the 2 m interval positioned immediately below the FFT-water interface. Additional details on the design and operation of this sampler are provided by Dompierre et al. (2017). Temperature and pH were measured immediately on the boat using a combined electrode (model 8172BNWP; Thermo Fisher Scientific, Waltham, MA, United States). The FFT samples were placed on ice and transferred to an on-site laboratory within 6 h. Sub-samples for microbial DNA extraction were collected into sterile 50 mL centrifuge tubes and stored at −20°C. The remaining FFT samples were refrigerated (4°C) for up to 12 h until analysis. Pore water was extracted by centrifugation (8500 × *g*, 30 min) and passed through 0.45 μm PES syringe filter membranes. Electrical conductivity was measured using a conductivity probe (model 013005MD; Thermo Fisher Scientific). Dissolved ΣH_2_S and NH_4_^+^ were quantified using the same methods described above for water cap sample analyses. Samples for dissolved SO_4_^2–^, NO_3_^–^, and NO_2_^–^ were transferred to HDPE bottles and refrigerated (4°C) until analysis by ion chromatography (Dionex ICS-2000, Thermo Fisher Scientific, Sunnyvale, CA, United States). Sub-samples for dissolved methane analysis were collected on the sampling boat immediately following collection of the FFT samples. For each dissolved methane sample, approximately 60 g of FFT was transferred into a 120 mL amber glass serum bottle, which was immediately crimp-sealed using a butyl rubber stopper. The samples were frozen to prevent ongoing methane production and subsequently thawed and placed on a shaker table at ambient temperature for 24 h before analysis. The headspace gas concentrations were quantified on a gas chromatograph fitted with a thermal conductivity detector (490 micro GC, Agilent Technologies, Santa Clara, CA, United States). Dissolved gas concentrations were determined using gravimetric water contents and established partitioning coefficients.

### Microbial DNA Extraction

Water cap microbial cells were collected by filtering ∼1.5 L water through 0.22 μm Rapid-Flow sterile disposable filters (Thermo Fisher Scientific) and stored at −20°C until the DNA extraction. Genomic DNA was extracted from the water cap filter samples using the DNeasy PowerWater DNA Isolation Kit (Qiagen, Hilden, Germany) and stored at −20°C for downstream analyses. A total of 18 samples were analyzed using microbial 16S rRNA gene amplicon sequencing (V4 region), and a subset of 11 were sequenced for metagenomics analyses (shotgun sequencing, Supplementary Table S1). Genomic DNA was extracted from ca. 0.5 g of each FFT sample using the FastDNA Spin Kit for Soil (MP Biomedical, Irvine, CA, United States), and stored at −20°C. The samples were homogenized in the cell disruption step to enhance extraction efficiency (Fast Prep 120, Thermo Scientific Savant, Waltham, MS, United States). The manufacturer protocol was modified to increase DNA quality and quantity. These modifications included repeating: (i) the cell disruption step two times and pooling resulting product before adding the binding matrix solution; (ii) the ethanol wash step three times to enhance removal of organics; and (iii) the elution step two times with DNA-free water to obtain 100–150 μL of DNA solution. DNA yield was confirmed with a Qubit 2.0 Fluorometer (Thermo Fisher Scientific) using a Qubit High Sensitivity dsDNA Assay Kit (Thermo Fisher Scientific) and DNA quality was assessed using an Epoch Microplate Spectrophotometer with Take3 plate (BioTek, Winooski, VT, United States).

### Microbial 16S rRNA Gene Amplicon Sequencing

Identical primers and variable region were used to characterize the microbial community structure in water and FFT genomic DNA samples; the high-throughput amplicon sequencing protocol is briefly summarized as follows. The variable region 4 of the 16S rRNA gene was amplified by PCR from the purified DNA, using Illumina adapted primers (Bartram et al., 2011) following standard protocols of the Earth Microbiome Project (Caporaso et al., 2011, 2012). The primer set 515f (5′-GTGYCAGCMGCCGCGGTAA-3′) and 806r (5′-GGACTACNVGGGTWTCTAAT-3′) was used to amplify the V4 region of the bacterial and archaeal 16S rRNA gene (Bates et al., 2010). The PCR reaction protocol was as follows: 50 ng of DNA as template, initial denaturing at 98°C for 5 min, 35 cycles of denaturing at 98°C for 30 s, annealing at 50°C for 30 s and extension at 72°C for 30 s, followed by final extension at 72°C for 10 min (Bates et al., 2010). For the water cap microbial community, PCR products were checked by gel electrophoresis and positive amplicons were sequenced using the Illumina MiSeq platform at Farncombe Metagenomics Facility at McMaster University (Hamilton, Canada). All amplicons were normalized using the SequalPrep normalization kit (Thermo Fisher Scientific) and sequenced with the Illumina MiSeq platform. Sequence reads were filtered and trimmed using Cutadapt (minimum quality score of 30 and a minimum read length 100 bp; Martin, 2011). Sequence variants were then resolved from the trimmed raw reads using a sample inference pipeline DADA2 and reported as sequence variants (Callahan et al., 2016). Sequence variant tables were merged to combine all information from separate Illumina runs. Bimeras were removed along with sequences identified as mitochondria or chloroplasts, and taxonomy was assigned using the SILVA database version 132. Sequences belonging to Eukaryota, chloroplasts and mitochondria were removed. For the FFT samples, amplicon sequencing was conducted using the Illumina MiSeq platform at the University of Calgary. The same V4 region of bacterial and archaeal 16S rRNA gene as the water cap samples (515f/806r) was amplified and sequenced. The PCR reactions were as follows: initial denaturing at 94°C for 3 min, 24 cycles of denaturing at 94°C for 45 s, annealing at 50°C for 60 s and extension at 72°C for 90 s, and final extension at 72°C for 10 min. Sequence reads were processed using the mothur bioinformatics software package MiSeq standard operating procedure (Schloss et al., 2009; Kozich et al., 2013). Chimeras were identified and removed using the VSEARCH algorithm (Rognes et al., 2016). Taxonomy was assigned using the SILVA database version 132.

### Water Cap Metagenomic Sequencing, *de novo* Assembly, and Genome Binning

Library construction and sequencing were performed at the Farncombe Metagenomics Facility at McMaster University (Hamilton, Canada). Microbial DNA samples (up to 1 μg) were fragmented using the Covaris S220 Ultrasonicator (Covaris, Woburn, MA, United States) in snap-cap microtubes. Parameters for 500 bp shearing with 50 μL input were: 175W PIP, 5% duty factor, 200 cpb, 35 s. Dual-indexed shotgun libraries were prepared with the NEBNext Ultra DNA Library Prep Kit for Illumina (New England Biolabs, Beverly, MA, United States). The libraries were quantitated by quantitative PCR, using primers complementary to the distal ends of the Illumina adaptors. Illumina’s PhiX v3 control library was used to generate a standard curve. Quantified libraries were pooled in equimolar amounts and sequenced using the Illumina HiSeq 1500 platform (Rapid v2 chemistry with onboard cluster generation, 151 bp paired-end reads). Raw data were processed with HCS v2.2.58 (RTA v1.18.64). File conversion and demultiplexing were performed with CASAVA v1.8.2 allowing 1 mismatch in the indexes. Raw reads were filtered to remove adapters and PhiX/Illumina sequencing contaminants, and then trimmed using the sickle software (Joshi and Fass, 2011) to remove low quality bases/reads. Quality reads from all samples was individually assembled using IDBA_UD (Peng et al., 2012) with kmer-set of “20, 40, 60, 80, 100, 120, 140.” The protein-coding genes were predicted from those scaffolds with a minimum length of 1000 bp using Prodigal (Hyatt et al., 2010), and annotated by searched using Usearch (Edgar, 2010) against the KEGG, UniRef100 and Uniprot databases. Also, 16S rRNA and tRNA genes were predicted from those scaffolds with HMM search (Brown et al., 2015) and tRNAscanSE (Lowe and Eddy, 1997), respectively. For coverage calculation, the corresponding quality reads were mapped to the scaffolds using Bowtie2 (Langmead and Salzberg, 2012) with default parameters, then the coverage was calculated as the total mapped bases divided the length of the scaffold. Genome binning was performed using MetaBAT (Kang et al., 2015) based on the coverage and tetranucleotide frequency (TNF) of each scaffold with default parameters. The initial genome bins obtained from MetaBAT were subsequently imported to ggKbase^1^, and manually modified based on taxonomic information of genes on scaffolds.

### Microbial Growth Rates (dRep and iRep)

All modified genome bins were evaluated by CheckM for genome completeness and contaminations (Parks et al., 2015). Representative genome bins were selected with dRep for genomes with a completeness ≥50% and contamination ≤10% (Olm et al., 2017). The growth rate of microorganisms in endemic Base Mine Lake communities was assessed using the index of replication (iRep) which determines microbial growth activity based on sequencing read coverage pattern (Brown et al., 2016) within representative bins.

### Phylogenetic Analyses

Phylogenetic trees of the 16S rRNA gene and selected functional genes, both derived from the shotgun sequencing data, were constructed based on the maximum-likelihood method using MEGA (v7.0.26) (Kumar et al., 2016). Reference sequences for the 16S rRNA gene and functional genes of different microorganisms were obtained using the SILVA (version 132) and IMG databases, respectively. Relevant nucleic acid or amino acid sequences were aligned using ClustalW in MEGA and the phylogenetic trees were created using Tamura-Nei model with 1000 bootstrap iterations.

### DNA Sequence Accession Numbers

The water cap microbial 16S rRNA gene amplicon sequences, as well as microbial genomes obtained from metagenomics are deposited at NCBI under BioProject PRJNA552483. The FFT microbial 16S rRNA gene sequences were deposited in the European Nucleotide Archive; study accession number PRJEB32633.

## Results

### Physico- and Geochemistry

The physicochemical and geochemical features of water cap and FFT samples from August of 2015 and 2016 are summarized in Supplementary Table S1. The water cap was thermally stratified with circumneutral pH. Oxygen concentrations decreased with depth, reaching <3% saturation at the FFT-water interface (9.0 m; Figure 1C and Supplementary Table S1), and becoming euxinic within the FFT. The highest level of dissolved aqueous methane concentration (∼70 μM) in the water cap was detected at the lower hypolimnetic zone. In the underlying FFT, methane was present at concentrations up to two orders of magnitude higher, reaching 3.6 mM at ∼2 m below the FFT-water interface (Supplementary Table S1). Nitrate, nitrite and ammonia were detectable throughout the water cap in both years, which were with higher concentrations in 2015 compared to 2016. FFT had higher ammonia concentrations (700∼1100 μM) than the water cap, while nitrite was non-detectable and nitrate was barely detected (∼1.3 μM). Sulfate concentrations were high and homogeneous in the water cap for both summers (1.5–2.6 mM), while dissolved ΣH_2_S was only detected at the anoxic FFT-water interface (∼1.24 μM) and in the underlying, shallow (from the FFT water interface to 2 m below) FFT zone (∼2.56 μM; Supplementary Table S1). TOC/DOC concentrations showed no evident depth dependence, ranging between 303∼458 mg/L and 43∼58 mg/L respectively (Supplementary Table S1).

### Microbial Community Analyses

**FIGURE 2:**
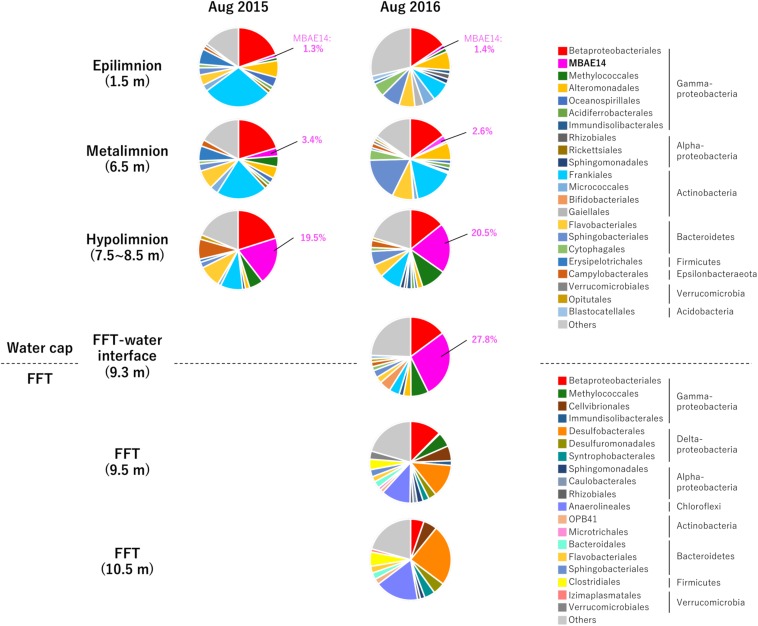
Relative abundances (%) detected by 16S rRNA gene amplicon sequencing of the 33 most abundant microbial orders for the three Base Mine Lake water cap stratified region depths (1.5, 3.5, and 7.5–8.5 m depth; 2015 and 2016); the FFT water interface (2016 only) and two depths within the FFT (9.5 and 10.5 m within the FFT; 2016 only). Numbers identify the percentages of MBAE14 (the major *amo*/*pmo* gene carrier) observed in water cap samples (0% in FFT).

**FIGURE 3:**
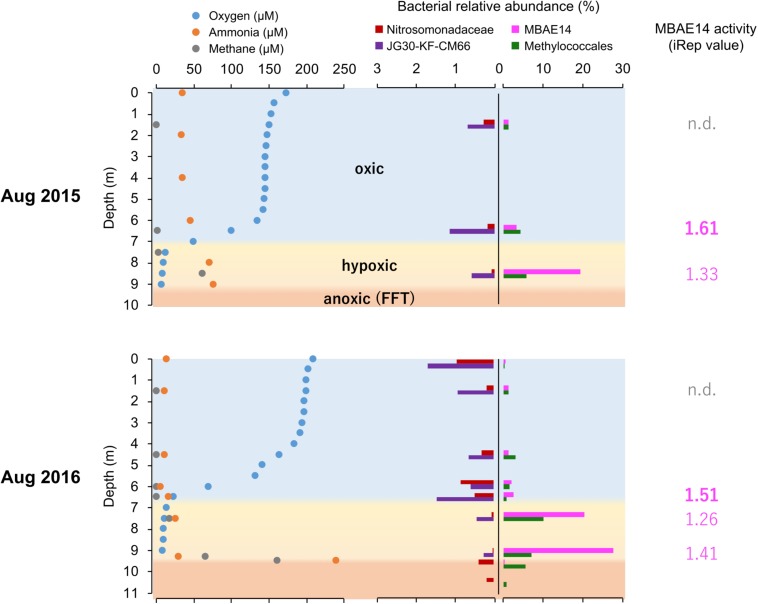
Summarized dissolved oxygen, ammonia and methane concentrations (μM) profiles, relative abundances (%) of putative nitrifiers and methanotrophs, as well as activity values based on iRep for MBAE14 in the Base Mine Lake water column in 2015 (above) and 2016 (below). Profiles of dissolved oxygen (blue), ammonia (orange) and methane (gray) depict oxic (light blue), hypoxic (<0.5 mg/L = ∼16 μM dissolved oxygen, yellow) and anoxic (<0.1 mg/L = ∼3 μM dissolved oxygen, orange) zones. Relative abundances of typical AOB Nitrosomonadaceae (red), putative nitrite-oxidizing Chloroflexi JG30-KF-CM66 (purple), putative AOB MBAE14 (pink), and methanotroph Methylococcales (green) in the Base Mine Lake water cap as well as in the FFT zones (only 2016) are shown in different colors. n.d., not detected.

Relative sequence variant abundances of each microbial class and order sequenced from the Base Mine Lake water cap as well as from three depths within the underlying FFT layer are summarized in Figure 2 and Supplementary Table S2. The common AOB Nitrosomonadaceae (Betaproteobacteriales; the SILVA database version 132 implements the Genome Taxonomy Database taxonomy system, which proposes that the class Betaproteobacteria is reclassified as an order within the class Gammaproteobacteria; Parks et al., 2018) was detected in all water cap samples collected from both summers, although in low abundance (<1%), with the highest abundances associated with the upper, more oxygenated water cap regions (Figure 3 and Supplementary Table S2). Intriguingly, an unclassified gammaproteobacterial group “MBAE14” was the most abundant microbial order detected in the low oxygen (<5% saturation) hypolimnetic and FFT-water interface zones in 2016 (20.5% at 7.5 m, 27.8% at 9.3 m), and ranked as the second most abundant order at 8.5 m in the water cap in 2015 (19.5%), with Betaproteobacteriales the most dominant group (20.1%). However, it was not detected in the underlying FFT (Figures 2, 3 and Supplementary Table S2). The known methanotroph Methylococcales was present throughout the water cap with higher abundance in the hypolimnetic zones (up to 10%), as well as right below the FFT-water interface (5.7%) but was not detected deeper within the FFT. Frankiales (<29%), Sphingobacteriales (<17%), and Flavobacteriales (<9%) dominated the oxic portion of the water cap, while Desulfobacterales (<24%) and Anaerolineales (<17%) dominated the anoxic FFT zone (Figure 2 and Supplementary Table S2).

### Metagenomics Survey for Microbial Functional Genes

Ammonia monooxygenase (*amo*ABC) and particulate methane monooxygenase (*pmo*ABC) genes were detected throughout the entire water column, revealing widespread genomic metabolic potential for nitrification and methanotrophy. Subsequent genome-reconstruction showed that these *amo*/*pmo* genes were encoded in genomes of the unclassified gammaproteobacterial group MBAE14 observed to be highly abundant in the hypolimnetic and the FFT-water interface zones (Figure 2), as were the methanotroph Methylococcales family (*Methylobacter*, *Methylovulum, Methylocaldum*), and an unclassified Burkholderiaceae (full 16S rRNA gene sequence was not obtained). Further, genes for hydroxylamine oxidoreductase (*hao*), the marker gene for the second step of ammonia-oxidation (oxidizing hydroxylamine to nitrite), was also detected in all analyzed metagenomic samples, but associated only with the MBAE14 group genome. Partial *amo* and *hao* genes from genomes of a typical AOB Nitrosomonadaceae (Betaproteobacteriales) were detected in the metalimnetic and hypolimnetic water cap samples for both summers, but with very low relative abundances. Genes for nitrite oxidoreductase (*nxr*AB, nitrite oxidation) were detected in a genome of an unclassified Chloroflexi group “JG30-KF-CM66,” and that genome was detectable in all analyzed samples. Collectively, these results indicate that nitrification within the Base Mine Lake water cap is likely associated with a novel unclassified gammaproteobacterial group MBAE14, aerobic AOB Nitrosomonadaceae, the nitrite-oxidizing Chloroflexi, as well as potentially Methylococcales and uncertain Burkholderiaceae though the latter are more likely methanotrophs (no *hao* genes detected).

### Replication Rates of Potential Nitrifiers in Water Column

The iRep analyses (replication rates, i.e., “activity”) were retrieved for the potential nitrifiers in the Base Mine Lake water cap, when a given genome has a minimum coverage of 5X in the sample (methods). Thus, iRep values for the *amo*/*pmo* carriers MBAE14 and Methylococcales (*Methylobacter* and another unassigned species) are only reported for the metalimnion and hypolimnion of the water cap, whereas the activity of Nitrosomonadaceae as well as Chloroflexi was not possible to determine from all samples. MBAE14 activity was relatively high in the metalimnion (1.61 in 2015 and 1.51 in 2016) compared to the hypolimnion (1.33 in 2015, 1.26–1.41 in 2016; Figure 3), while Methylococcales showed an opposite trend (1.82 and 1.45 in the hypolimnion for 2015 and 2016 respectively; 1.64 in the metalimnion in 2015).

### Genomic Characterization for the Gammaproteobacterial Group MBAE14

**FIGURE 4:**
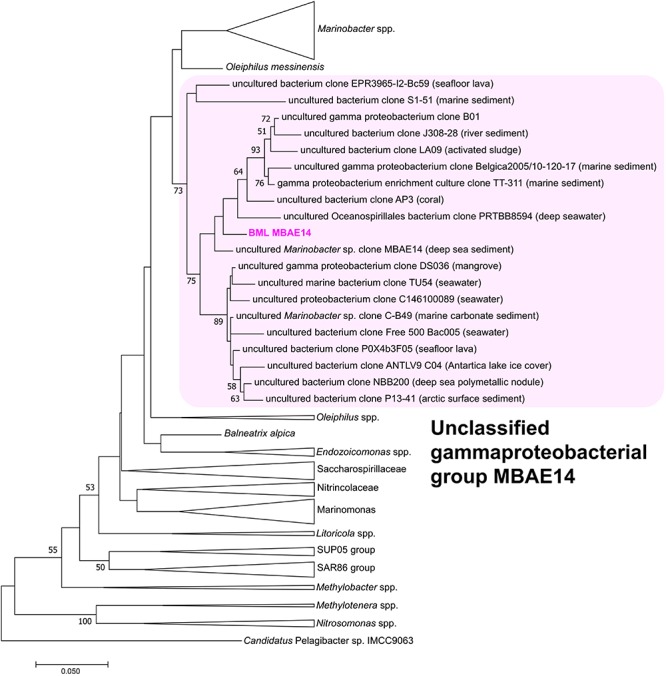
Maximum-likelihood phylogenetic tree of 16S rRNA gene of the BML MBAE14 and phylogenetically-related bacterial groups. The tree was rooted with 16S rRNA gene of *Candidatus* Pelagibacter sp. IMCC9063 (SAR11 group, Alphaproteobacteria). The tree was created with 1000 bootstrap iteration (values below 50% are not reported). The origins of each uncultured bacterial clone belonging to the MBAE14 group are reported in parentheses.

A full length 16S rRNA gene (1535 bp) was identified in the genome of MBAE14. From phylogenetic analyses, the MBAE14 organism found in Base Mine Lake (“BML MBAE14”) showed low 16S rRNA gene sequence homologies (<94.8%) and taxonomically the organism is located in a unclassified, uncultured gammaproteobacterial clade MBAE14 (SILVA database 132; Figure 4 and Table 1). The closest cultured bacterial groups to MBAE14 are represented by Oceanospirillales (*Oleiphilus* spp., Family Nitrincolaceae, *Marinomonas* spp., Family Saccharospirillaceae) and *Marinobacter* spp. (Alteromonadales).

**TABLE 1:** Characterization of the potential metabolic capabilities for BML MBAE14 compared to phylogenetically-related bacterial groups as well as known methanotrophic and nitrifying bacterial/archaeal groups.

**Organism**	**Taxonomy**			**% homology with BML MBAE14**	**Group description**	**Presence of functional genes (# in IMG genome database)**									
	**Class**	**Order**	**Family**			**Nitirification**			**Denitrification**					**CO_2_-fixation**	
						***amo* (*pmo*)**	***hao***	***nxr*A/*nar*G**	***nap*A**	***nir*K**	***nir*S**	***nor*B**	***nos*Z**	**Rubis CO**	***acl***
BML MBAE14	Gammaproteobacteria	MBAE14		–	Bacterium in Base Mine Lake	**+**	**+**	**+**	–	**+**	–	**+**	**+**	–	–
MBAE14 group				<94.8	Uncultured marine group	No genome available in database									
*Oleiphilus* spp.		Oceanospirillales	Oleiphilaceae	<90.0	Marine heterotrophs	0/29	0/29	13/29	8/29	0/29	14/29	13/29	12/29	0/29	0/29
*Marinobacter* spp.		Alteromonadales	Marinobacteraceae	<90.0	Marine heterotrophs	0/85	0/85	**60/85**	11/85	7/85	30/85	**55/85**	**59/85**	0/85	0/85
Nitrincolaceae		Oceanospirillales		<89.7	Marine heterotrophs	0/29	0/29	8/29	2/29	1/29	2/29	8/29	8/29	3/29	0/29
Saccharospirillaceae				<89.6	Marine heterotrophs	0/20	0/20	1/20	5/20	1/20	1/20	3/20	3/20	0/20	0/20
*Marinomonas* spp.			Marinomonadaceae	<87.9	Marine heterotrophs	0/26	0/26	0/26	2/26	0/26	0/26	0/26	0/26	4/26	0/26
*Pseudomonas stutzeri*		Pseudomonadales	Pseudomonadaceae	<88.4	Heterotrophic AOB	0/33^∗^	0/33^∗^	**25/33**	**29/33**	12/33	**30/33**	**29/33**	**29/33**	1/33	0/33
*Methylococcus* spp.		Methylococcales	Methylococcaceae	<87.5	Type X Methanotrophs	**3/3**	**3/3**	0/3	0/3	0/3	0/3	**3/3**	0/3	**3/3**	0/3
*Methylobacter* spp.				<87.3	Type I Methanotrophs	**18/18**	5/18	**10/18**	0/18	7/18	0/18	5/18	0/18	0/18	0/18
*Nitrosococcus* spp.		Nitrosococcales	Nitrosococcaceae	<84.9	Autotrophic AOB	**7/7**	**7/7**	0/7	0/7	**6/7**	0/7	**7/7**	0/7	**6/7**	0/7
*Nitrosomonas* spp.		Betaproteobacteriales	Nitrosomonadaceae	<80.9	Autotrophic AOB	**51/52**	**47/52**	0/52	0/52	**44/52**	0/52	**35/52**	0/52	**52/52**	0/49
*Nitrosospira* spp.				<79.9	Autotrophic AOB	**31/31**	**31/31**	0/31	0/31	**28/31**	0/31	14/31	0/31	**31/31**	0/31
*Alcaligenes faecalis*			Burkholderiaceae	<78.9	Heterotrophic AOB	0/9^∗^	0/9^∗^	0/9	0/9	**9/9**	0/9	**9/9**	**9/9**	**5/9**	0/9
*Paracoccus pantotrophus*	Alphaproteobacteria	Rhodobacterales	Rhodobacteraceae	<77.1	Heterotrophic AOB	0/5^∗^	0/5^∗^	**5/5**	**5/5**	0/5	**5/5**	**5/5**	**5/5**	**5/5**	0/5
Nitrospirae (comammox)	(Candidatus) Nitrospira			<75	Comammox	**4/4**	**4/4**	**4/4**	1/4	**4/4**	**2/4**	0/4	0/4	1/4	**4/4**
*Nitrosopumilus* spp. (Thaumarchaeota)	Nitrososphaeria	Nitrosopumilales	Nitrosopumilaceae	<75	Autotrophic AOA	4/9	0/9	0/9	0/9	4/9	0/9	0/9	0/9	0/9	0/9

*Presence of functional genes is shown in gray (present in > 30% of the reference genomes of each bacterial group in IMG database; bold, >50%). ^∗^Putative genes (not homologous) and enzymes were previously sequenced or purified. amoA (pmoA), ammonia monooxygenase (ammonia → hydroxylamine) (methane → methanol); hao, hydroxylamine oxidoreductase (hydroxylamine → nitrite); nxrA/narG, napA, nitrite oxidoreductase/nitrate reductase (nitrite ←→ nitrate); nirK, nirS, nitrite reductase (nitrite → nitric oxide); norB, nitric oxide reductase (nitric oxide → nitrous oxide); nosZ, nitrous oxide reductase (nitrous oxide → nitrogen); RubisCO, ribulose-biosphosphate carboxylase (key enzyme for CO_2_-fixation by Calvin-Benson-Bessham cycle); acl, ATP-citrate lyase (key enzyme for CO_2_-fixation by reductive TCA cycle).*

**FIGURE 5:**
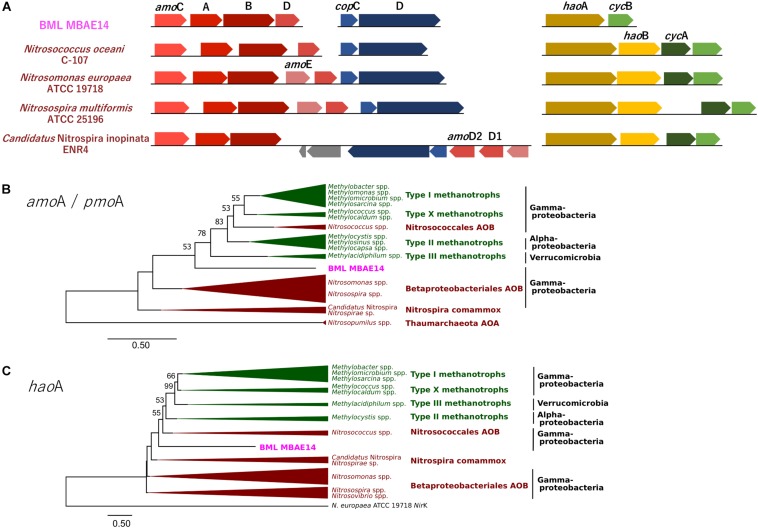
**(A)** Gene clusters of *amo* and *hao* genes encoded by BML MBAE14 and other known AOB. **(B,C)** Maximum-likelihood phylogenetic trees of the functional genes *amo*A/*pmo*A **(B)** and *hao*A **(C)** found in BML MBAE14 (pink) and typical groups of ammonia oxidizers (brown) and methanotrophs (green). The tree was rooted with the genes of *Nitrosopumilus* spp. (Thaumarchaeota AOA) *amo*A gene **(B)** and *Nitrosomonas europaea* ATCC19718 *Nir*K gene **(C)** and created with 1000 bootstrap iteration (values below 50% are not reported).

**FIGURE 6:**
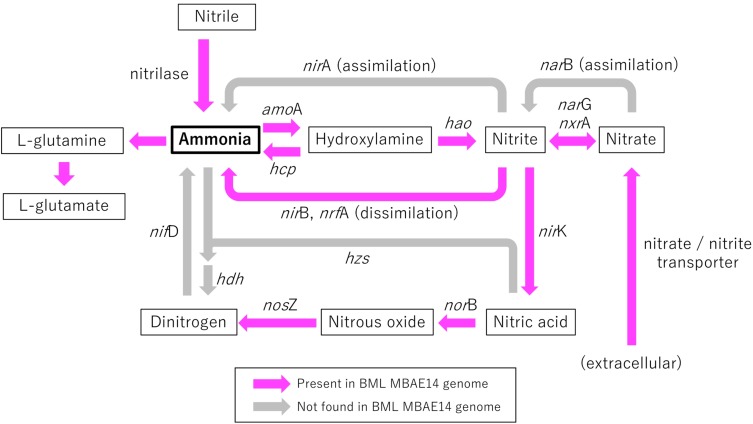
Schematic model of functional genes involved in nitrogen metabolism encoded in the genome of BML MBAE14. Arrows indicate functional gene pathways, which are present (pink) or absent (i.e., inferred; gray) in the genome. *amo*A, ammonia monooxygenase (EC 1.14.99.39); *hcp*, hydroxylamine reductase (EC 1.7.99.1); *hao*, hydroxylamine oxidoreductase (EC 1.7.2.6); *nir*A, (assimilatory) nitrite reductase (EC 1.7.7.1); *nir*B/*nrf*A, (dissimilatory) nitrite reductase (EC1.7.1.15, 1.7.2.2); *nar*G/*nxr*A, (dissimilatory) nitrate reductase/nitrite oxidoreductase (EC 1.7.5.1, 1.7.99.-); *nar*B, (assimilatory) nitrate reductase (EC 1.7.7.2); *nir*K, (NO-forming) nitrite reductase (EC 1.7.2.1); *nor*B, nitric oxide reductase (EC 1.7.2.5); *nos*Z, nitrous oxide reductase (EC 1.7.2.4); *hzs*, hydrazine synthase (EC 1.7.2.7); *hdh*, hydrazine dehydrogenase (EC 1.7.2.8); *nif*D, nitrogenase (EC 1.18.6.1).

A genome (96.11% completeness, 3.15 Mbp) of the BML MBAE14 was reconstructed from metagenomic data (information is summarized in Supplementary Table S3). The BML MBAE14 genome encodes gene clusters including *amo*ABC (EC 1.14.99.39) and *hao* (EC 1.7.2.6) for nitrification (and potentially methanotrophy), which is widely conserved in other known AOB and comammox (Figure 5A). These *amo* and *hao* genes were distantly related to those of the known autotrophic AOB, comammox, AOA as well as methanotrophs (Figures 5B,C). Interestingly, BML MBAE14 also possesses genes for the whole pathway of denitrification (NO_3_^–^/NO_2_^–^ → NO → N_2_O → N_2_) as well as a nitrate/nitrite transporter (Figure 6 and Table 1). The BML MBAE14 genome does not encode any known genes for CO_2_-fixation for autotrophic growth (such as Calvin-Benson-Bessham cycle, reductive TCA cycle and reductive acetyl-CoA pathway, Table 1). In addition, BML MBAE14 possesses gene sets related to glycolysis and fatty acid degradation, while encoding none of the functional genes for methanotrophic pathways (methanol → formaldehyde, carbon-fixation via RuMP cycle or serine cycle), suggesting they possess heterotrophic rather than methanotrophic capabilities.

## Discussion

### The Microbial Nitrogen Cycle Within a Manmade Oil Sands Mining Pit Lake

Niches for aerobic microbial nitrification are created by the relative availabilities of ammonia, oxygen and organic matter. Autotrophic, aerobic nitrifiers can compete for ammonia and oxygen with heterotrophs in ammonia-rich and organic matter-limited environments (Kuypers et al., 2018). However, an adequate supply of ammonia typically relies on ammonification from organic matter mineralization coupled to nitrate/nitrite reduction, which requires high organic matter loads (Giblin et al., 2013). The Base Mine Lake water cap exhibits specific conditions of an ammonia-rich and labile organic matter-limited environment (Foght et al., 2017), creating a niche capable of supporting the novel potential AOB group MBAE14, in the hypolimnetic and the FFT-water interface zones (Figures 2, 3 and Supplementary Table S2). While the Base Mine Lake water cap is characterized as brackish (approximately 400 mg/L chloride; White and Liber, 2018), the bacterial groups related to BML MBAE14 (Oceanospirillales and *Marinobacter* spp.) were previously reported only in marine environments (Gauthier et al., 1992; Cappello and Yakimov, 2010; Satomi and Fujii, 2014). Furthermore, although BML MBAE14 and some related groups (Oceanospirillales and *Marinobacter* spp.) are potentially capable of denitrification, none of these groups carries functional genes for methanotrophy and nitrification (Table 1; IMG genome database). Some known AOB are also capable of reducing nitrite to nitric acid, but none of the currently identified AOB encodes the full denitrification process to dinitrogen as observed in BML MBAE14, *Oleiphilus* spp., and *Marinobacter* spp. (Table 1 and Figure 6). Such combined nitrification-denitrification metabolism has been reported for many groups of heterotrophic AOB (Guo et al., 2013; Stein, 2016 and references therein). The genome insights on BML MBAE14 revealed here suggest this organism is a heterotrophic ammonia-oxidizer which conducts a combined nitrification and denitrification process, while relying on organic carbon for growth.

**FIGURE 7:**
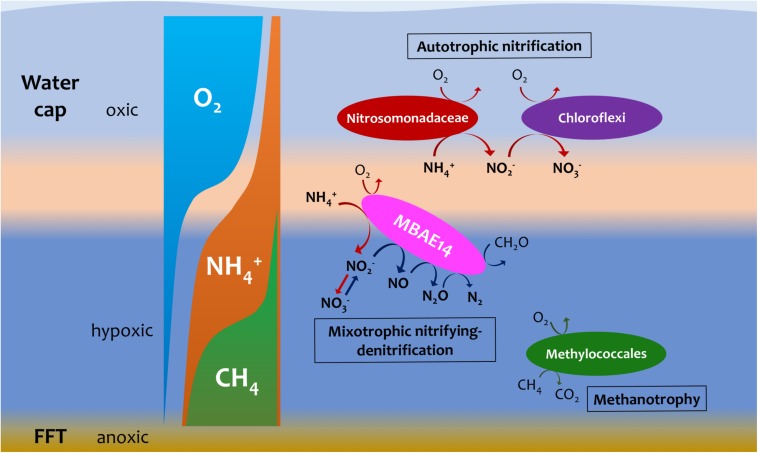
Hypothetic, schematic model summarizing nitrogen and methane biogeochemistry in the summer stratified Base Mine Lake water column (August 2015 and 2016 respectively). A novel gammaproteobacterial group MBAE14 (proposed mixotrophic nitrifying-denitrifier), thrives at the oxic/hypoxic zones of Base Mine Lake water cap separated from autotrophic aerobic nitrifiers (Nitrosomonadaceae and nitrite-oxidizing Chloroflexi). The metalimnetic oxygen-ammonia transition zone (light orange) is suggested as the hotspot for aerobic nitrification by different nitrifiers, while methanotrophy (conducted by Methylococcales) primarily occurs in the hypoxic lower hypolimnetic zones where elevated concentrations of dissolved methane were observed.

The putative heterotrophic AOB, BML MBAE14 group, and the autotrophic AOB Nitrosomonadaceae (Betaproteobacteriales) appear to be the key ammonia-oxidizers in Base Mine Lake, although the activities of eukaryotes in this system were not evaluated in our metagenomics analyses. Interestingly, these nitrifiers co-occur in time, but their abundances are spatially (depth) separated, presumably driven by the available dissolved oxygen, i.e., while the autotrophic nitrifiers (Nitrosomonadaceae and putative nitrite-oxidizing Chloroflexi) were more abundant within the shallower, more oxygenated epilimnetic and metalimnetic zones, MBAE14 dominated the community in the hypolimnetic region (2–2.5 meters above the FFT-water interface), where oxygen concentrations were lower (<16 μM = ∼0.5 mg/L; Figure 3). Our results are consistent with the dominance of heterotrophic AOB over autotrophic AOB under oxygen limiting concentrations (<1 mg/L) (van Niel et al., 1993; Kampschreur et al., 2009). Heterotrophic ammonia oxidation exhibits 100–1000 times slower rates compared to autotrophic ammonia oxidation (Kampschreur et al., 2009), and the spatial separation of these organisms observed here suggests geochemical conditions segregate autotrophic and heterotrophic ammonia oxidation pathways within the water cap. Although MBAE14 seems adapted to the hypolimnetic hypoxic zones, its growth (iRep inferred) activity was higher within the more oxygenated metalimnetic region than in the hypolimnion (Figure 3). The geochemical results indicate that the Base Mine Lake water cap metalimnetic region comprises opposing concentration gradients of ammonia and oxygen (Figures 1C, 3), suggesting a hotspot for microbial ammonia oxidation (Figure 7). In contrast, concentrations of the three N redox species barely changed above the metalimnion, suggesting microbial nitrification was less active in the upper water zone compared to the deeper zone, although ammonia was available (∼35 μM) even at the water surface (Figure 1C and Supplementary Table S1). Methanotrophy, on the other hand, mostly occurred within the lower hypolimnion, which is consistent with the abundance and activity of the methanotrophic Methylococcales (Figures 2, 3, 7) and the occurrence of dissolved aqueous methane above trace concentrations (i.e., >1 μM) only within the lower hypolimnetic waters (Figure 1C).

Oxygen appeared as a key factor for the distribution of MBAE14 as well as other nitrifiers. Indeed, the absence of any of these nitrifiers in the underlying anoxic FFT suggests these groups are not able to survive without the capability to carry out aerobic nitrification and were thus outcompeted by anaerobes. Expected anaerobic AOB (anammox) groups (i.e., Planctomycetes), as well as the functional genes for anaerobic ammonia oxidation (*hzs* and *hdh*) were not detected in samples from the Base Mine Lake water column. This result suggests their exclusion is potentially due to the limited nitrite supply, and/or the high bulk organic carbon content of Base Mine Lake water, as anammox pathways are preferentially observed in organic matter-limited environments (Kumar et al., 2017). These results reveal that the ammonia-rich oxic/hypoxic transition zone occurring within the Base Mine Lake hypolimnetic region of the water cap provides a unique nitrogen cycling niche required by this novel, putative heterotrophic nitrifier MBAE14.

### The Nitrogen/Carbon Utilization Potential of the MBAE14 Group

The well-studied aerobic nitrifiers which oxidize: (1) ammonia to nitrite belong to Gammaproteobacteria (*Nitrosomonas* and *Nitrosospira* in the order Betaproteobacteriales, and *Nitrosococcus*) and Thaumarchaeota (*Nitrosopumilus*); (2) nitrite to nitrate belong to Alphaproteobacteria (*Nitrobacter*), Gammaproteobacteria (*Nitrococcus*) and Deltaproteobacteria (*Nitrospina*); and (3) Nitrospira which are capable of completely oxidizing ammonia to nitrate (comammox). Heterotrophic ammonia oxidation has been reported in various prokaryotes and also eukaryotes (fungi and algae; Schmidt et al., 2003). Indeed, many heterotrophic AOB such as *Pseudomonas* spp., *Alcaligenes* spp., *Bacillus* spp., and *Paracoccus* spp., are known to conduct combined nitrification and aerobic denitrification (supposedly do not gain energy for growth through nitrification) in soils, sewage sludges, bioreactors and wastewater (Stein, 2016 and references therein). This process has been attracting interest due to its potentially important contribution to the nitrogen cycle in natural environments such as soils (Banerjee and Siciliano, 2012), as well as for its efficiency for removal of nitrogen species which can be applied to wastewater treatments (Joo et al., 2005; Duan et al., 2015). Importantly, homologous genes of *amo* and *hao* were not found in genomes of these known heterotrophic AOB (such as *Pseudomonas stutzeri*, *Alcaligenes faecalis*, and *Paracoccus pantotrophus*, Table 1), suggesting these marker genes are conserved only among the autotrophic nitrifiers. The potential AOB and HAO enzymes detected from heterotrophic AOB are sequentially and structurally different from those of autotrophic AOB, suggesting heterotrophic AOB are incapable of energy-generating nitrification (Bothe et al., 2000). The presence of *amo* and *hao* homologous genes in the genome of BML MBAE14 (Figures 5B,C and Table 1) infers that BML MBAE14 possibly utilizes ammonia as an additional or sole energy source, conducting mixotrophic (i.e., chemolitho-heterotrophic) growth in addition to denitrification. However, we could not detect the *hao*B gene in the MBAE14 genome, which is well conserved in genomes of autotrophic AOB (IMG database), implying a different gene/enzyme might be involved. Many autotrophic AOB are also capable of denitrification in addition to energy-generating nitrification, known as the nitrifier denitrification pathway; this metabolism usually happens under severe hypoxic conditions (Poth and Focht, 1985; Shaw et al., 2006) similar to the conditions of the Base Mine Lake hypolimnion where BML MBAE14 was detected. Further, other well-known AOB such as *Nitrosomonas* have been reported to grow mixotrophically, indicating high functional flexibility of AOB (Hommes et al., 2003). Indeed, the nitrifier denitrification process is thermodynamically favorable (ΔG^0^ = −360 kJ) (Broda, 1977) and eventually generates comparable energy to organotrophic denitrification on acetate in oxygen-limited environments (ΔG^0^ = −398 kJ), indicating the ecological likelihood and relevance of this pathway (Lam and Kuypers, 2011).

To the best of our knowledge, no cultivation or genomic characterization for the other members belonging to MBAE14 group have been conducted prior to this study. Indeed, the ecology of this group is poorly understood and few studies/environments report their presence; previous occurrences are limited to Arctic deep sea sediments (Li et al., 2015), coastal subsurface sediments (Gupta et al., 2009), and surface seawater where strains were recovered by high-throughput cultivation (Yang et al., 2016). Interestingly, none of these environments are characterized by high ammonia concentrations, however, Li et al. (2015) found the Arctic deep sea sediment enriched with ammonia-oxidizing Thaumarchaeota known to play a major role in ammonia oxidation in oxygen-limited environments (Yan et al., 2012). Although this finding may suggest that marine and freshwater/brackish members of the MBAE14 group present a different affinity for ammonia, it is clear that further research is needed to characterize the diversity and environmental distribution of this MBAE14 group which appears to expand possible nitrogen cycling pathways. Our integrated geochemical-metagenomic approach presents the first insights into the metabolic potential of the MBAE14 group, and further cultivation-based characterization followed by stable isotope analyses and/or transcriptomics approach are expected to fully constrain the nitrogen/carbon utilization capabilities of this group.

## Conclusion

Engineered systems provide opportunities to discover novel microorganisms driving putative and yet to be identified metabolic processes harbored in their unusual physicochemical and geochemical contexts. Here, we report on a currently unclassified gammaproteobacterial group MBAE14 from Base Mine Lake, the first full-scale demonstration pit lake in the Athabasca oil sands region, that appears capable of both nitrification and denitrification, adding to our understanding of the nitrogen cycle. Our combined metagenome and geochemical results point to spatial segregation of nitrogen cycling by MBAE14 within the water cap, dependent on geochemical conditions. Water cap depth dependent opposing gradients of ammonia and oxygen create specialized conditions enabling MBAE14 to carry out aerobic, chemolithotrophic ammonia oxidation at the metalimnetic oxycline where oxygen concentrations are higher, whilst dominating the hypolimnetic hypoxic zones (<0.5 mg/L dissolved oxygen), where it conducts denitrification converting nitrate/nitrite to dinitrogen associated with the mixotrophic nitrifier denitrification pathway. The limitation of labile organic carbon, occurrence of depth dependent geochemical niches and flexibility of MBAE14 nitrogen metabolic pathways, collectively enable this organism to thrive in co-occurrence with other aerobic heterotrophs under higher oxygen concentrations in the metalimnion, as well as with methanotrophs under low oxygen conditions in the hypolimnion. Our results highlight the need for more metagenomics microbial functional gene surveys in these ecologically dynamic systems to better inform our understanding of important biogeochemical cycles.

## Data Availability Statement

The datasets generated for this study can be found in the PRJNA552483 and PRJEB32633.

## Author Contributions

JM and LW designed the experiments and prepared the manuscript and figures, incorporating input from all authors. JM, GJ, SR, JM, and ML were responsible for field sampling, geochemical analysis, microbial DNA extraction, and 16S rRNA sequence analysis and interpretation (JM and GJ for the water cap; SR, JM, and ML for the underlying FFT layers). GS provided input on field sampling methods and geochemical analysis. L-XC and JB contributed to the metagenomic analysis and interpretation (*de novo* assembly, genome binning, and iRep analysis).

## Conflict of Interest

The authors declare that the research was conducted in the absence of any commercial or financial relationships that could be construed as a potential conflict of interest.

## Footnotes

1 https://ggkbase.berkeley.edu/
